# A Core–Shell Pt–NiSe@NiFe-LDH Heterostructure for Bifunctional Alkaline Water Splitting

**DOI:** 10.3390/molecules30234654

**Published:** 2025-12-04

**Authors:** Shanshan Li, Yanping Guo, Ziqi Wang, Depeng Zhao, Rui Guo, Qingzhong Gao, Zhiqiang Zhang

**Affiliations:** 1 School of Chemical Engineering, Liaoning University of Science and Technology, Anshan 114051, China; 2 School of New Energy, Shenyang Institute of Engineering, Shenyang 110136, China

**Keywords:** oxygen evolution reaction (OER), hydrogen evolution reaction (HER), water splitting, AEMWE

## Abstract

The escalating global energy crisis has intensified the demand for sustainable hydrogen production through electrochemical water splitting. Herein, we report a novel oxygen-vacancy-rich bifunctional electrocatalyst, Pt-NiSe@NiFe-LDH-Ov, synthesized via a facile electrodeposition and reduction method. It demonstrates exceptional performance, requiring low overpotentials of 280 mV for the HER and 344 mV for the OER to achieve current densities of 50 and 100 mA cm^−2^, respectively, in 1.0 M KOH. When employed for overall water splitting, the system requires a cell voltage of only 1.878 V to reach 50 mA cm^−2^. Notably, in an anion exchange membrane water electrolyzer (AEMWE), the performance shows significant enhancement with increasing operating temperature (20 to 60 °C), particularly at high current densities (>200 mA cm^−2^), highlighting its excellent thermal adaptability. The superior activity is attributed to the synergistic effect between the Pt-NiSe and NiFe-LDH interfaces and the abundant oxygen vacancies, which collectively enhance charge transfer and optimize the adsorption of reaction intermediates.

## 1. Introduction

Facing the dual challenges of the accelerating depletion of fossil fuels and the continuous deterioration of ecosystems, building a green and sustainable energy supply system has become an urgent global issue [1,2,3]. Against this backdrop, hydrogen energy, with its clean, efficient, and recyclable characteristics, is regarded as one of the key pathways to drive the transition of the energy structure [4,5]. Among the various technologies, water electrolysis represents a highly promising green hydrogen production method, in which the core processes are the hydrogen evolution reaction (HER) at the cathode and the oxygen evolution reaction (OER) at the anode [6,7,8]. Compared with HER, which involves only a two-electron transfer, OER is much slower in kinetics due to its complex four-electron/proton-coupled transfer process [9,10]. This reaction entails the evolution of multiple intermediates and requires overcoming high-energy barriers, often resulting in a significant overpotential [11]. Noble-metal catalysts such as Ru and IrO_2_ can effectively lower the OER overpotential, while Pt exhibits excellent catalytic activity for HER [12,13]. However, achieving high catalytic efficiency while reducing the reliance on noble metals remains one of the critical challenges in the field of electrocatalysis [14].

Non-noble metal catalysts mainly include transition metal oxides, phosphides, selenides, and sulfides [15,16]. Among them, transition metal selenides have attracted increasing attention in recent years due to their excellent electrocatalytic activity and good stability [17,18]. However, pure-phase materials such as NiSe_2_, FeSe_2_, and CoSe_2_ generally suffer from poor conductivity, selenium leaching, and surface oxidation, which limit their practical applications [19,20,21]. To address these issues, researchers have commonly employed strategies such as constructing heterostructures, element doping, alloying, or compositing with carbon materials to enhance catalytic performance [22]. For example, Li et al. [23] successfully fabricated NiSe@La-FeNi_3_ nanorods using a hydrothermal method combined with electrodeposition. Their study revealed that the introduction of La atoms significantly strengthened the electronic interactions between NiSe and FeNi_3_, thereby facilitating charge transfer. As a result, the catalyst exhibited outstanding bifunctional activity for both the HER and the OER, requiring overpotentials of only 34 mV (HER) and 238 mV (OER) at current densities of 10 mA cm^−2^ and 50 mA cm^−2^, respectively. Similarly, Zhong et al. [24] designed a carbon-coated NiSe/FeO_x_ heterostructure. Density functional theory (DFT) calculations demonstrated that the carbon layer not only effectively suppressed the leaching and agglomeration of NiSe and FeO_x_ during the reaction but also accelerated electron transfer through dual interfacial effects, thereby markedly enhancing OER performance, with an overpotential as low as 265 mV at 50 mA cm^−2^. These findings suggest that introducing heteroatoms or constructing heterogeneous interfaces can effectively modulate the electronic structure of catalysts, thereby significantly improving their HER and OER activities.

This study successfully synthesized Pt-modified Pt-NiSe@NiFe-LDH-Ov catalysts by employing NiSe as a sacrificial template through a two-step electrodeposition combined with the NaBH_4_ reduction method. The introduction of Pt atoms effectively enhanced the electronic coupling interaction between NiSe and NiFe-LDH, significantly improving the charge transfer capability. Electrochemical tests demonstrated that the catalyst exhibited excellent bifunctional catalytic performance in 1 M KOH electrolyte: an overpotential of only 280 mV was required to achieve a current density of 50 mA cm^−2^ for the HER, while an overpotential of 344 mV was sufficient to reach 100 mA cm^−2^ for the OER. These findings definitively confirm the synergistic effects of noble metal atom doping and heterostructure engineering in enhancing electrocatalytic activity, providing new design strategies and experimental foundations for developing efficient and stable water electrolysis catalysts.

## 2. Results and Discussion

**Figure 1:**
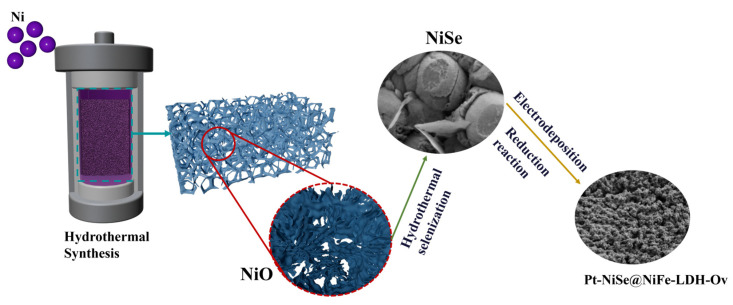
Synthetic illustration of spherical Pt-NiSe@NiFe-LDH-Ov samples.

As illustrated in Figure 1, the Pt-NiSe@NiFe-LDH-Ov catalyst was synthesized via a two-step electrodeposition process followed by chemical reduction. First, NiSe nanospheres were successfully synthesized on nickel foam by a simple hydrothermal method. Subsequently, NiSe nanospheres served as a self-sacrificial template, on which NiFe-LDH nanosheets were deposited through one-step electrodeposition to form a NiSe@NiFe-LDH heterostructure. In the second electrodeposition step, Pt atoms were uniformly incorporated into the NiSe@NiFe-LDH framework, yielding the Pt-NiSe@NiFe-LDH precursor. Finally, chemical reduction in KBH_4_ solution generated abundant oxygen vacancies, resulting in the construction of the Pt-NiSe@NiFe-LDH-Ov heterostructure. This catalyst features an optimized electronic structure and excellent intrinsic catalytic activity, while maintaining a hierarchical nanostructure favorable for mass transport and active-site exposure, thereby providing the structural basis for its superior electrocatalytic performance.

**Figure 2:**
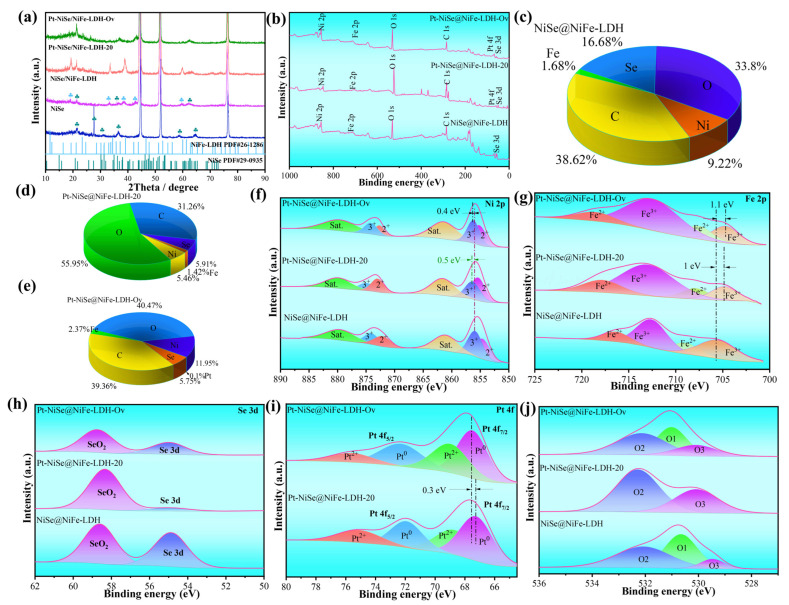
(**a**) XRD patterns. (**b**) The full XPS spectrum of the NiSe@NiFe-LDH, Pt-NiSe@NiFe-LDH-20, and Pt-NiSe@NiFe-LDH-Ov samples. XPS spectra of elemental composition for (**c**) NiSe@NiFe-LDH, (**d**) Pt-NiSe@NiFe-LDH-20, and (**e**) Pt-NiSe@NiFe-LDH-Ov samples. The high-resolution XPS of (**f**) Ni 2p, (**g**) Fe 2p, (**h**) Se 3d, (**i**) Pt 4f, and (**j**) O 1s.

The crystal structure and phase composition of the as-prepared materials were characterized by X-ray diffraction (XRD). As shown in Figure 2a, sharp diffraction peaks at 2θ values of 21.40°, 27.64°, 30.96°, 36.68°, 58.78°, and 64.48° correspond to the (023), (111), (041), (044), (224), and (158) planes of NiSe (PDF#29-0935), confirming the good crystallinity of the NiSe catalyst. After the first electrodeposition of NiFe-LDH on NiSe, additional peaks appeared at 18.94°, 33.22°, 38.70°, 42.60°, and 59.64°, which are assigned to the (105), (118), (2,0,10), (2,0,13), and (220) planes of NiFe-LDH (PDF#26-1286), indicating the successful formation of the NiSe@NiFe-LDH heterostructure. Following the second electrodeposition step, the intensity of NiFe-LDH peaks increased, suggesting the successful incorporation of Pt atoms into the NiFe-LDH lattice. After KBH_4_ reduction, the XRD patterns of the obtained samples still retained the characteristic peaks of both NiSe and NiFe-LDH. Collectively, the XRD results confirm the successful synthesis of NiSe, NiSe@NiFe-LDH, Pt-NiSe@NiFe-LDH-20, and Pt-NiSe@NiFe-LDH-Ov catalysts.

To further elucidate the evolution of the electronic structure, X-ray photoelectron spectroscopy (XPS) was employed to investigate the surface chemical states of NiSe@NiFe-LDH, Pt-NiSe@NiFe-LDH-20, and Pt-NiSe@NiFe-LDH-Ov catalysts. As displayed in Figure 2b, the survey spectrum of NiSe@NiFe-LDH exhibits distinct signals of Ni, Se, Fe, O, and C. After Pt incorporation, clear Pt signals emerged in Pt-NiSe@NiFe-LDH-20 and Pt-NiSe@NiFe-LDH-Ov, confirming the successful doping of Pt into the catalyst structure. Elemental composition analysis (Figure 2c) shows that Ni, Se, Fe, O, and C account for 9.22%, 16.68%, 1.68%, 33.81%, and 38.62%, respectively, in NiSe@NiFe-LDH. Upon Pt doping (Figure 2d,e), the relative content of O increased significantly, while that of Ni, Se, and Fe decreased, further corroborating the successful introduction of Pt and its influence on surface chemistry.

In the Ni 2p spectrum (Figure 2f), the peaks at 854.7 eV and 872.2 eV are assigned to Ni^2+^ 2p_3/2_ and 2p_1/2_, while those at 856.0 eV and 874.1 eV correspond to Ni^3+^ 2p_3/2_ and 2p_1/2_ [25]. Satellite peaks at 861.3 eV and 879.8 eV further confirm the coexistence of Ni^2+^ and Ni^3+^ [26]. After Pt doping, the higher electronegativity of Pt led to a decrease in the electron cloud density of Ni, resulting in a positive shift in the Ni 2p binding energy [27]. Specifically, Pt-NiSe@NiFe-LDH-20 and Pt-NiSe@NiFe-LDH-Ov exhibited shifts of 0.5 eV and 0.4 eV, respectively, indicating strong electronic interactions between Pt and Ni that promoted charge redistribution.

The Fe 2p spectrum (Figure 2g) shows characteristic Fe 2p_3/2_ and Fe 2p_1/2_ peaks in all samples, indicating the coexistence of Fe^2+^ and Fe^3+^ [28]. Compared with the undoped sample, the Fe 2p binding energies of Pt-NiSe@NiFe-LDH-20 and Pt-NiSe@NiFe-LDH-Ov shifted positively, suggesting that some Pt atoms may have partially substituted Fe sites, inducing Fe → Pt electron transfer and thereby enhancing electronic coupling within the material [29,30]. As shown in Figure 2h, the Se 3d spectrum consists of two main peaks corresponding to Se–Ni bonding states and oxidized Se in SeO_2_ [31]. From NiSe@NiFe-LDH to Pt-NiSe@NiFe-LDH-Ov, slight shifts in Se 3d binding energies were observed, particularly after KBH_4_ reduction, implying that the coordination environment of Se was modified due to changes in the electronic density of surrounding metal centers [32]. The Pt 4f spectrum (Figure 2i) reveals that the intensity of Pt^0^ peaks increased significantly in Pt-NiSe@NiFe-LDH-Ov, indicating that KBH_4_ reduction promoted partial reduction of Pt species [33]. Moreover, the narrowed Pt 4f peak width suggests a more uniform chemical environment and better dispersion of Pt, which benefits the accessibility of catalytically active sites [34]. The O 1s spectrum (Figure 2j) further reveals surface oxygen evolution. Analysis of the O 1s XPS spectrum indicates that Pt–NiSe@NiFe-LDH-Ov exhibited the lowest intensity of the lattice oxygen (M–O) peak at 530.1 eV and the highest relative proportion of the oxygen vacancy (Ov) peak at 531 eV compared with Pt–NiSe@NiFe-LDH-20 [35,36]. This observation demonstrates that KBH_4_-induced treatment generated surface oxygen defects and hydroxyl groups, which optimized the surface chemical properties of the material and effectively promoted the adsorption and activation of water molecules. These modifications substantially enhanced the catalytic activity of the material in both the HER and OER.

**Figure 3:**
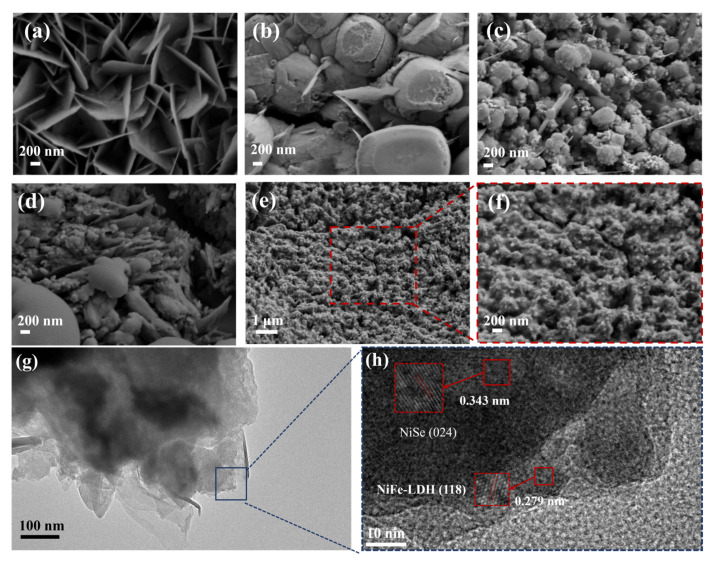
SEM images of (**a**) NiO, (**b**) NiSe, (**c**) NiSe@NiFe-LDH, (**d**) Pt-NiSe@NiFe-LDH-20, and (**e**,**f**) Pt-NiSe@NiFe-LDH-Ov. (**g**) TEM images of Pt-NiSe@NiFe-LDH-Ov samples. (**h**) HRTEM images of Pt-NiSe@NiFe-LDH-Ov samples.

The surface morphologies of the catalysts were investigated using scanning electron microscopy (SEM). As shown in Figure 3a, NiO exhibited nanosheets formed by the interlaced stacking of flake-like structures. After hydrothermal selenization, the nanosheet morphology was transformed into uniform nanospheres (Figure 3b), which substantially increased the electrochemically active surface area, thereby benefiting catalytic activity. For NiSe@NiFe-LDH (Figure 3c), the nanosphere morphology of NiSe remained intact, but the surface became rougher, confirming the successful deposition of NiFe-LDH nanosheets and the formation of a NiSe@NiFe-LDH heterostructure. Upon Pt electrodeposition (Figure 3d), the particle size of the nanospheres decreased, and the surface roughness increased, exposing more active sites. SEM images of Pt-NiSe@NiFe-LDH-Ov at different magnifications (Figure 3e,f) show that the nanospheres further decreased in size and became more uniformly distributed after KBH_4_ reduction, resulting in denser coverage and higher surface-active site density, which is favorable for enhancing catalytic performance. The structural characteristics of the sample were analyzed using Transmission Electron Microscopy (TEM). Figure 3g,h presents the TEM and High-Resolution Transmission Electron Microscopy (HRTEM) images of the Pt-NiSe@NiFe-LDH-Ov sample, respectively. As observed in the images, the sample exhibits a rough nanospherical morphology. Furthermore, the lattice fringes with interplanar spacings of 0.343 nm and 0.279 nm correspond to the (024) plane of the NiSe sample and the (118) plane of the NiFe-LDH sample, respectively.

**Figure 4:**
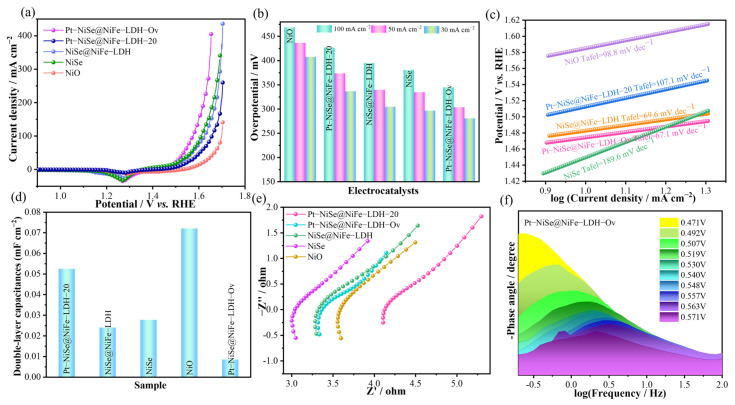
OER performances of the electrocatalysts in 1.0 M KOH. (**a**) LSV curves. (**b**) Overpotential bar plots. (**c**) Tafel plots. (**d**) Double-layer capacitance. (**e**) Nyquist plots. (**f**) Bode plots.

The OER performance of the catalysts in 1 M KOH electrolyte was systematically evaluated by linear sweep voltammetry (LSV). As shown in Figure 4a, Pt-NiSe@NiFe-LDH-Ov exhibited superior OER activity. In terms of overpotential comparison (Figure 4b), this catalyst required only 280 mV at 30 mA cm^−2^, much lower than NiO (408 mV), NiSe (295 mV), NiSe@NiFe-LDH (304 mV), and Pt-NiSe@NiFe-LDH-20 (341 mV). Remarkably, even at a high current density of 100 mA cm^−2^, the overpotential remained as low as 344 mV, demonstrating excellent catalytic performance under high current density. Tafel slope analysis (Figure 4c) further confirmed favorable reaction kinetics, with Pt-NiSe@NiFe-LDH-Ov exhibiting a slope of only 67.1 mV dec^−1^, much lower than NiO (98.8 mV dec^−1^) and NiSe (189.6 mV dec^−1^), as well as Pt-NiSe@NiFe-LDH-20 (107.1 mV dec^−1^), and only slightly higher than NiSe@NiFe-LDH (69.6 mV dec^−1^). These results indicate enhanced charge-transfer capability and accelerated kinetics. The electrochemically active surface area (ECSA) was estimated by double-layer capacitance (C_dl_). As shown in Figure 4d, Pt-NiSe@NiFe-LDH-Ov exhibited a C_dl_ value of 0.00849 mF cm^−2^. The reduction in C_dl_ after KBH_4_ treatment can be attributed to by-products covering some active sites. Electrochemical impedance spectroscopy (EIS) analysis (Figure 4e) shows that Pt-NiSe@NiFe-LDH-Ov displayed a much smaller semicircle diameter, indicating significantly reduced charge-transfer resistance and confirming that heterostructure construction and oxygen-vacancy engineering effectively enhanced electrocatalytic performance [37]. A further Bode phase analysis was performed within the potential range of 0.471–0.571 V (Figure 4f). As the potential increased to 0.519 V, a phase peak emerged in the low-frequency region. This peak shifted toward higher frequencies and its magnitude increased with rising potential, indicating the onset of the OER, an accelerated charge transfer rate, and enhanced capacitive behavior at the active interface [38]. The absence of additional peaks in the medium-to-high frequency region suggests that the reaction is governed by charge transfer kinetics without significant diffusion limitations [39]. This behavior is attributed to the introduction of Pt, which optimizes the interfacial charge transport between NiSe and NiFe-LDH, thereby significantly enhancing the electrocatalytic activity of the material. As shown in Table 1, the OER performance of Pt−NiSe@NiFe−LDH−Ov is significantly superior to that of the other comparative samples.

**Figure 5:**
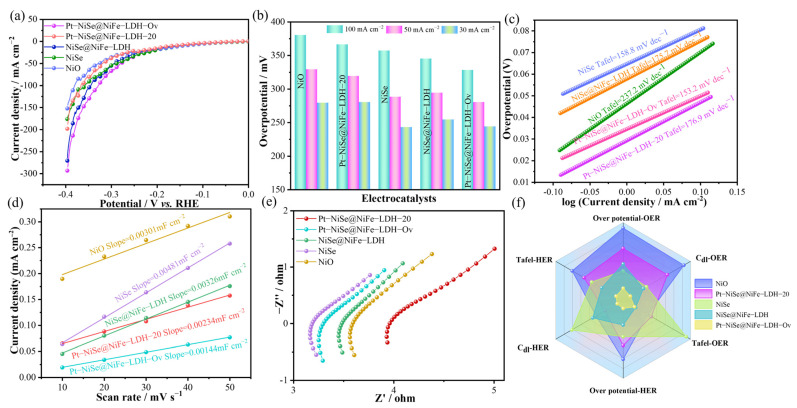
HER performances of the electrocatalysts in 1.0 M KOH. (**a**) LSV curves. (**b**) Overpotential bar plots. (**c**) Tafel plots. (**d**) Double-layer capacitance. (**e**) Nyquist plots. (**f**) Radar chart.

To verify its bifunctional catalytic potential, the HER performance of Pt-NiSe@NiFe-LDH-Ov was evaluated in a 1 M KOH electrolyte. As shown in Figure 5a, Pt-NiSe@NiFe-LDH-Ov exhibited significantly enhanced HER activity compared with other reference catalysts. The overpotential comparison (Figure 5b) revealed that at a current density of 50 mA cm^−2^, Pt-NiSe@NiFe-LDH-Ov required only 280 mV, which is 40, 9, 14, and 39 mV lower than that of NiO, NiSe, NiSe@NiFe-LDH, and Pt-NiSe@NiFe-LDH-20, respectively. Tafel slope analysis (Figure 5c) further showed that Pt-NiSe@NiFe-LDH-Ov achieved a slope of 153.2 mV dec^−1^, markedly lower than NiO (237.2 mV dec^−1^), NiSe (158.8 mV dec^−1^), NiSe@NiFe-LDH (175.7 mV dec^−1^), and Pt-NiSe@NiFe-LDH-20 (176.9 mV dec^−1^), confirming its superior HER kinetics. As illustrated in Figure 5d, the C_dl_ value of Pt-NiSe@NiFe-LDH-Ov was 0.00144 mF cm^−2^, consistent with the trend observed in the OER process, where a reduction in C_dl_ was attributed to surface active sites being partially blocked by by-products formed during KBH_4_ reduction. Impedance spectra (Figure 5e) revealed a catalytic activity ranking of Pt-NiSe@NiFe-LDH-20 > Pt-NiSe@NiFe-LDH-Ov > NiSe@NiFe-LDH > NiSe > NiO, which aligned with the C_dl_ results, further supporting the notion that KBH_4_ reduction led to partial surface site coverage. A radar plot (Figure 5f) highlighted the excellent bifunctional activity of Pt-NiSe@NiFe-LDH-Ov for both HER and OER, demonstrating its potential as an efficient dual-function electrocatalyst.

**Figure 6:**
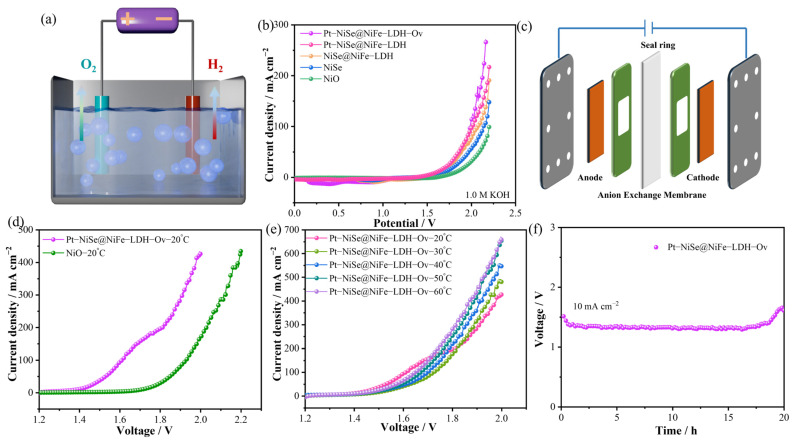
(**a**) Schematic illustration of overall water splitting. (**b**) LSV curves of the catalyst in 1 M KOH. (**c**) Schematic diagram of the AEM electrolyzer. (**d**) AEMWE Polarization curves of electrolytic cells with different catalysts at 20 °C. (**e**) Polarization curves of electrolytic cells with Pt-NiSe@NiFe-LDH-Ov. (**f**) Cycling stability of the Pt-NiSe@NiFe-LDH-Ov sample.

To systematically evaluate the practical application of the prepared catalyst in water splitting, an electrolyzer was constructed using Pt-NiSe@NiFe-LDH-Ov as both anode and cathode (Figure 6a). In 1.0 M KOH electrolyte, the LSV curves (Figure 6b) showed that at 50 mA cm^−2^, Pt-NiSe@NiFe-LDH-Ov required only 1.878 V, which is significantly lower than NiO (2.089 V), NiSe (1.968 V), and NiSe@NiFe-LDH (1.883 V), and also superior to Pt-NiSe@NiFe-LDH-20 (1.858 V). At 150 mA cm^−2^, the cell voltage was 2.055 V, outperforming Pt-NiSe@NiFe-LDH-20 (2.129 V), indicating excellent activity under high current density. Given its exceptional catalytic activity and stability in 1 M KOH, we constructed an anion exchange membrane water electrolyzer (AEMWE) to assess the practical potential of Pt-NiSe@NiFe-LDH-Ov (Figure 6c). The AEMWE employed 1.0 M KOH as the electrolyte, maintained at an operating temperature of 20 °C. Preheated ultrapure water was continuously circulated through the system at a flow rate of 50 mL min^−1^. The polarization curve was measured with a cut-off voltage set at 2.2 V, and the operational stability was systematically evaluated by monitoring the voltage change over time. When configured as a bifunctional electrode with dimensions of 1.0 cm × 1.0 cm, the Pt-NiSe@NiFe-LDH-Ov-based AEMWE required a cell voltage of only 1.609 V to achieve a current density of 100 mA cm^−2^ (Figure 6d), which is significantly lower than the 1.928 V required by the conventional NiO‖NiO configuration. Temperature-dependent tests (Figure 6f) further revealed that the electrolysis performance continuously improved as the operating temperature increased from 20 °C to 60 °C, with the advantage becoming more pronounced at higher current densities. Notably, it required only 1.923 V to operate stably at 500 mA cm^−2^. Furthermore, during a 20-h chronopotentiometric test at 10 mA cm^−2^ (Figure 6e), the cell voltage showed negligible degradation, robustly confirming the outstanding long-term stability of the catalyst. In summary, Pt-NiSe@NiFe-LDH-Ov demonstrates great potential as an efficient and stable bifunctional electrocatalyst.

## 3. Experimental Sections

### 3.1. Synthesis of NiSe Catalyst

NiO precursor was prepared via a hydrothermal method. Briefly, 1 mM Ni(NO_3_)_2_∙6H_2_O, 6 mM NH_4_F, and 12.5 mM urea were dissolved in 50 mL of deionized water and magnetically stirred at room temperature for 1 h to obtain a homogeneous precursor solution. The cleaned nickel foam and the precursor solution were then transferred into a 100 mL Teflon-lined autoclave and maintained at 120 °C for 6 h. After natural cooling to room temperature, the obtained samples were washed thoroughly with deionized water and ethanol, followed by drying in an oven at 60 °C for 12 h to yield NiO precursor-loaded nickel foam.

Subsequently, 0.5 g Se powder and 5 g NaOH were dissolved in 60 mL deionized water and stirred until homogeneous, followed by hydrothermal treatment at 180 °C for 12 h. The resulting solution, together with the NiO precursor, was then placed in a 100 mL autoclave and maintained at 140 °C for 5 h to obtain the NiSe catalyst.

### 3.2. Synthesis of NiSe@NiFe-LDH Catalyst

NiFe-LDH was electrodeposited on the NiSe catalyst using a standard three-electrode system, with NiSe as the working electrode, Ag/AgCl (saturated KCl) as the reference electrode, and a Pt foil as the counter electrode. The electrolyte consisted of 10 mM Ni(NO_3_)_2_∙6H_2_O and 10 mM Fe(NO_3_)_3_∙9H_2_O aqueous solution. Electrodeposition was conducted for 60 s to load NiFe-LDH. After electrodeposition, the samples were rinsed with deionized water and ethanol, followed by drying in a vacuum oven at 60 °C for 12 h to obtain the NiSe@NiFe-LDH catalyst.

### 3.3. Synthesis of Pt-NiSe@NiFe-LDH Catalyst

The Pt-NiSe@NiFe-LDH catalyst was synthesized by electrodeposition. The as-prepared NiSe@NiFe-LDH catalyst was immersed in 0.4 mM H_2_PtCl_6_ aqueous solution, and cyclic voltammetry (CV) was performed between −0.50 and 0.50 V vs. RH) at a scan rate of 5 mV s^−1^ in 1 M KOH. After 10, 20, and 30 CV cycles, the catalysts were denoted as Pt-NiSe@NiFe-LDH-10, Pt-NiSe@NiFe-LDH-20, and Pt-NiSe@NiFe-LDH-30, respectively.

### 3.4. Synthesis of Pt-NiSe@NiFe-LDH-Ov Catalyst

Finally, the Pt-NiSe@NiFe-LDH catalyst was treated with potassium borohydride (NaBH_4_) reduction to obtain the Pt-NiSe@NiFe-LDH-Ov catalyst.

## 4. Electrocatalytic Performance Evaluation

All electrocatalytic performances were measured using an IviumSoft electrochemical workstation. OER and HER tests were conducted in a three-electrode system with the as-prepared electrocatalysts as working electrodes and Hg/HgO as the reference electrode. Graphite rods and platinum sheets were used as counter electrodes for HER and OER, respectively, with scan rates of 5 mV s^−1^ and 2 mV s^−1^. The electrolytes consisted of a 1 M KOH solution (pH = 13.7). Both HER and OER potentials were converted to reversible hydrogen electrode (RHE) scale using the equation: E (vs. RHE) = E (vs. Hg/HgO) + 0.059 × pH + 0.098 V. Additionally, overall water splitting performance was evaluated in a two-electrode configuration with a scan rate of 5 mV s^−1^.

## 5. Material Characterization

The phase composition of the materials was analyzed by X-ray diffraction (XRD, 7000, Shimadzu), SEM (Gemini, 300-71-31) and TEM (FEI Tecnai F20).The surface elemental composition and chemical states were analyzed by X-ray photoelectron spectroscopy (XPS, ESCALAB250, Al K_α_ sources).

## 6. Conclusions

In this study, NiSe nanospheres were successfully synthesized via a one-step hydrothermal method, followed by the construction of a Pt-NiSe@NiFe-LDH-20 heterostructure through a two-step electrodeposition process. Subsequently, a borohydride reduction treatment was employed to generate oxygen-vacancy-rich Pt-NiSe@NiFe-LDH-Ov heterostructured catalysts for efficient alkaline electrocatalytic water splitting. The composition, chemical states, and microstructures of the catalysts were systematically characterized by XRD, XPS, and SEM, confirming the successful synthesis and unique structural features of the target materials. Benefiting from the introduction of oxygen vacancies and the construction of heterointerfaces, Pt-NiSe@NiFe-LDH-Ov exhibits outstanding bifunctional catalytic activity for both the HER and OER. In OER performance tests, the catalyst requires an overpotential of only 344 mV to deliver a current density of 100 mA cm^−2^, with a Tafel slope of 67.1 mV dec^−1^, indicating favorable reaction kinetics. In HER testing, the catalyst maintains a relatively low overpotential (280 mV) even at a high current density of 50 mA cm^−2^, demonstrating excellent cathodic catalytic capability. For overall water splitting, an electrolyzer assembled with Pt-NiSe@NiFe-LDH-Ov as both electrodes requires a cell voltage of only 1.878 V to achieve 50 mA cm^−2^ in 1.0 M KOH electrolyte. To further evaluate its practical application potential, the catalyst was integrated into an AEMWE device, where it achieved stable operation at 100 mA cm^−2^ with an electrolysis voltage of just 1.609 V. Moreover, temperature-dependent measurements in the range of 20–60 °C revealed continuously enhanced catalytic performance with increasing temperature, particularly under high current density conditions. This work demonstrates that the rational design of oxygen vacancies coupled with heterostructure engineering offers a promising strategy for developing efficient and durable non-precious-metal-based bifunctional electrocatalysts.

## Figures and Tables

**Table 1:** **Electrocatalytic performance of the samples**.

Catalysts	Overpotential	Tafel (mV dec^−1^)	Electrolyte	Ref.
MnSe@MWCNT	OER: 290 mV@10	54.76	1 M KOH	[40]
O-NiSe_180-12_@Ni/SS	OER: 290 mV@10	48	1 M KOH	[41]
FeSe_2_/NiSe_2_	OER: 316 mV@20	137	1 M KOH	[42]
MoCo–NiSe (8:1)	OER: 277 mV@10	139	1 M KOH	[43]
NiSe_2_-FeSe DHP	OER: 280 mV@10	58	1 M KOH	[44]
NiSe/NF-4	OER: 370 mV@100	95.3	1 M KOH	[45]
Co–MoS_2_	OER: 312 mV@10	—	1 M KOH	[46]
	HER: 297 mV@10			
Pt-NiSe@NiFe-LDH-Ov	OER: 344 mV@100	67.1	1 M KOH	our work
	OER: 280 mV@30			
	HER: 280 mV@10	153.2		

## Data Availability

The raw data supporting the conclusions of this article will be made available by the authors on request.
